# Features and Comparative Characteristics of Fucosylated Glycans Expression in Endothelial Glycocalyx of Placental Terminal Villi in Patients with Preeclampsia Treated with Different Antihypertensive Regimens

**DOI:** 10.3390/ijms242115611

**Published:** 2023-10-26

**Authors:** Marina M. Ziganshina, Galina V. Kulikova, Kamilla T. Muminova, Alexander I. Shchegolev, Ekaterina L. Yarotskaya, Zulfiya S. Khodzhaeva, Gennady T. Sukhikh

**Affiliations:** 1 Laboratory of Clinical Immunology, National Medical Research Center for Obstetrics, Gynecology, and Perinatology Named after Academician V.I. Kulakov of the Ministry of Health of the Russian Federation, Oparina Str. 4, 117997 Moscow, Russia; sekretariat@oparina4.ru; 2 Department of Perinatal Pathology, National Medical Research Center for Obstetrics, Gynecology, and Perinatology Named after Academician V.I. Kulakov of the Ministry of Health of the Russian Federation, Oparina Str. 4, 117997 Moscow, Russia; gvkulikova@gmail.com (G.V.K.); ashegolev@oparina4.ru (A.I.S.); 3 High Risk Pregnancy Department, National Medical Research Center for Obstetrics, Gynecology, and Perinatology Named after Academician V.I. Kulakov of the Ministry of Health of the Russian Federation, Oparina Str. 4, 117997 Moscow, Russia; kamika91@mail.ru (K.T.M.); zkhodjaeva@mail.ru (Z.S.K.); 4 Department of International Cooperation, National Medical Research Center for Obstetrics, Gynecology, and Perinatology Named after Academician V.I. Kulakov of the Ministry of Health of the Russian Federation, Oparina Str. 4, 117997 Moscow, Russia; inter_otdel@mail.ru; 5 Department of Obstetrics, Gynecology, Perinatology and Reproductology, Faculty for Postgraduate and Advanced Training of Physicians, I.M. Sechenov First Moscow State Medical University of the Ministry of Health of the Russian Federation (Sechenov University), 119991 Moscow, Russia

**Keywords:** preeclampsia, placenta, fucosylated glycans, antihypertensive therapy, dopegyt, cordaflex, endothelial glycocalyx

## Abstract

Antihypertensive therapy is an essential part of management of patients with preeclampsia (PE). Methyldopa (Dopegyt^®^) and nifedipine (Cordaflex^®^) are basic medications of therapy since they stabilize blood pressure without affecting the fetus. Their effect on the endothelium of placental vessels has not yet been studied. In this study, we analyzed the effect of antihypertensive therapy on the expression of fucosylated glycans in fetal capillaries of placental terminal villi in patients with early-onset PE (EOPE) and late-onset PE (LOPE), and determined correlation between their expression and mother’s hemodynamic parameters, fetoplacental system, factors reflecting inflammatory response, and destructive processes in the endothelial glycocalyx (eGC). A total of 76 women were enrolled in the study: the comparison group consisted of 15 women with healthy pregnancy, and the main group comprised 61 women with early-onset and late-onset PE, who received one-component or two-component antihypertensive therapy. Hemodynamic status was assessed by daily blood pressure monitoring, dopplerometry of maternal placental and fetoplacental blood flows, and the levels of IL-18, IL-6, TNFα, galectin-3, endocan-1, syndecan-1, and hyaluronan in the blood of the mother. Expression of fucosylated glycans was assessed by staining placental sections with AAL, UEA-I, LTL lectins, and anti-Le^Y^ MAbs. It was found that (i) expression patterns of fucosylated glycans in eGC capillaries of placental terminal villi in EOPE and LOPE are characterized by predominant expression of structures with a type 2 core and have a similar pattern of quantitative changes, which seems to be due to the impact of one-component and two-component antihypertensive therapy on their expression; (ii) correlation patterns indicate interrelated changes in the molecular composition of eGC fucoglycans and indicators reflecting changes in maternal hemodynamics, fetoplacental hemodynamics, and humoral factors associated with eGC damage. The presented study is the first to demonstrate the features of placental eGC in women with PE treated with antihypertensive therapy. This study also considers placental fucoglycans as a functional part of the eGC, which affects hemodynamics in the mother–placenta–fetus system.

## 1. Introduction

Preeclampsia (PE) belongs to a group of severe complications of pregnancy, with the placenta playing a central role in its pathogenesis. Two main clinical phenotypes of PE have been described. Impaired trophoblast invasion causes early-onset PE (EOPE) with manifestation before 34 weeks; the leading pathogenetic factor is placental. The maternal factor, i.e., inherent maternal cardiovascular dysfunction, causes the development of late-onset PE (LOPE), with manifestation starting from 34 weeks [1]. LOPE comprises around 80% to 95% of all PE cases, while EOPE, although less common, is associated with a higher maternal morbidity and fetal growth restriction (FGR) or neonatal mortality rates [2]. Both clinical phenotypes are characterized by ischemic-hypoxic placental lesions of varying severity. Recent studies of PE pathogenesis demonstrated that EOPE is associated with a proinflammatory placental state, while LOPE is associated with systemic inflammation in the mother. Both subtypes are associated with maternal endothelial dysfunction [3,4,5]. Endothelial dysfunction together with systemic inflammatory response cause maladaptation of maternal and placental hemodynamics, which clinically show as changes in the blood flow in the fetoplacental system and impaired regulation of systemic blood pressure (BP), and affect obstetric and perinatal outcomes, as well as long-term outcomes for mother and child [2].

The range of antihypertensive medications used for treatment of arterial hypertension (AH) in pregnant women is limited because, along with the decrease in the BP in the mother, the drugs must not cause any negative impact on the fetus. One-component antihypertensive therapy with the use of Dopegyt^®^ (methyldopa), and two-component therapy—a combination of Dopegyt^®^ and, most often, Cordaflex^®^ (nifedipine)—are the common regimens of antihypertensive therapy currently used in clinical practice in Russia, since they meet both requirements mentioned above [6,7].

The mechanisms of effects of Dopegyt^®^ (central-acting agent) and Cordaflex^®^ (a long-acting calcium channel blocker) are well known [8,9,10]. However, their effects on the endothelial glycocalyx (eGC), the key structure that determines vascular “health” and regulates vascular tone by stimulating production of the endogenous vasodilator, nitric oxide (NO), have not yet been studied. Pro-inflammatory stimuli are among the main factors affecting the eGC, causing its shedding, which affects the mechanosensitivity of endothelial cells, changing their biochemical response, including the synthesis of NO [11]. Shedding causes exfoliation of the upper layer of the eGC, exposing hidden glycan structures [12]. Therefore, the production of pro-inflammatory cytokines and the expression of fucoglycans, either implicit or present as terminal groups, are interconnected, since both of these reflect the severity of the inflammatory response and resulting destruction of the eGC. Our previous studies showed that signs of endothelial dysfunction persist in EOPE and LOPE under both antihypertensive therapy regimens. However, a positive effect of therapy on eGC stabilization and reduction of its “desquamation”, confirmed by a decrease in the content of structural components of the eGC in the mother’s blood, was found only in the late PE [13]. We have also demonstrated high production of proinflammatory cytokines in the background of both antihypertensive regimens, especially in LOPE [14]. In this study, we evaluated the effect of antihypertensive therapy on the expression of fucosylated glycans of the eGC in the fetal capillaries of placental terminal villi in patients with EOPE and LOPE. Fucosylated glycans were chosen as target glycans because of their important role in the placenta [15,16,17], in particular, their involvement in angiogenesis and intercellular communication [18]. The study was focused on fucoglycans detected by UEA-I, LTL, and AAL lectins. According to previous publications, these lectins bind specifically to terminal clustered fucose residues linked to α1,6 and α1,3 to N-acetylglucosamine, or to fucose-containing glycans with core type 1 or core type 2 [15]. The study determined correlation patterns for each PE phenotype and antihypertensive therapy regimen; this made it possible to determine a correlation between the expression of fucosylated glycans, hemodynamic parameters of the mother and the fetoplacental system, and factors reflecting the inflammatory response and destructive processes in the eGC.

## 2. Results

### 2.1. Clinical Characteristics of the Patients Included in the Study

The patients of the study groups, according to the rank analysis of variances using the Kruskal–Wallis H test (Table 1) for “age” and “BMI” parameters, were comparable and did not significantly differ in the parameters known as the cofounders of the study. Mean values of MAP (mean arterial pressure) and DAP (diastolic arterial pressure), along with the characteristics of newborns, showed a pattern of significantly higher blood pressure and lower neonatal body weight and Apgar score in patients with pregnancy complicated by PE, regardless of gestational age and type of antihypertensive therapy, compared to control. Pairwise comparison with the Mann–Whitney U test of the groups of PE patients under two antihypertensive therapy regimens showed that patients with early- and late-onset PE who received one- and two-component therapy did not differ significantly in gestational age at delivery. This made it possible to compare clinical and laboratory data of the groups of PE patients receiving different therapy regimens at similar gestational ages.

### 2.2. Description of the Specificity Profile of Lectins Used for Placental Tissue Staining

**Figure 1:**
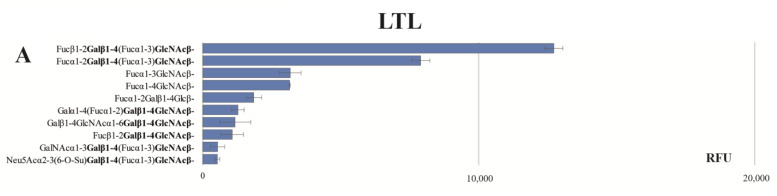

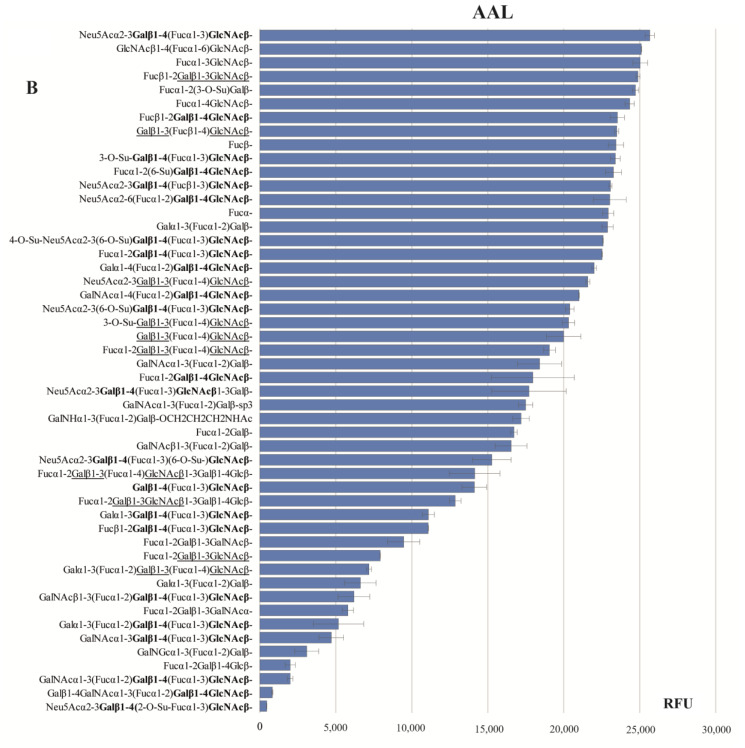
Carbohydrate specificity of plant lectins LTL (**A**) and AAL (**B**) determined on a glycochip. Core type 1 is underlined, core type 2 is in bold. X-axis indicates median of relative fluorescence units (RFUs) of six replicates of glycan. The median deviation was measured as an interquartile range. A signal with fluorescence intensity. RFU scale range is 0–65,535 RFU.

The carbohydrate structures that bind to AAL and LTL lectins were characterized in detail using a glycochip and are presented in Figure 1. The characterization of UEA-I lectin specificity was carried out previously on a chip of similar format [19]. A more detailed review of the carbohydrate specificity of these three lectins was reported by Shilova et al., 2023 [20]. The results of lectin binding to microarray glycans showed LTL interaction with fucosylated oligosaccharides that have an N-acetyllactosamine Galβ1-4GlcNAc core (type 2 carbohydrate structures), including Le^Y^ as well as H type 6 (Figure 1A). The specificity of LTL appeared to be similar to that of UEA-I described earlier [16]. UEA-I is also known to bind to type 2 carbohydrate structures, but the spectrum of structures is broader and includes, in addition to Le^Y^ and H type 6 glycans, the H type 2 disaccharide and other oligosaccharides. Despite sharing common structures in their specificity profiles, these two lectins have individual differences in the glycans that bind to them. Lectin AAL interacts with a broader spectrum of fucosylated oligosaccharides than LTL and UEA-I: both N-acetyllactosamine core and isolactosamine core (Galβ1-3GlcNAc, type 1 structures), as well as terminal fragments of blood group H, A, and B antigens (Figure 1B). Characterization of the specificity of commercial anti-Le^Y^ MAbs had been carried out by Ziganshina et al., 2021 [21]. Notably, anti-Le^Y^ MAbs was found to bind only to the difucosylated Le^Y^ oligosaccharide (Fucα1-2Galβ1-4(Fucα1-3)GlcNAcβ-R) [21].

### 2.3. Expression of Fucosylated Glycans in the Endothelium of Placental Terminal Villi

**Figure 2:**
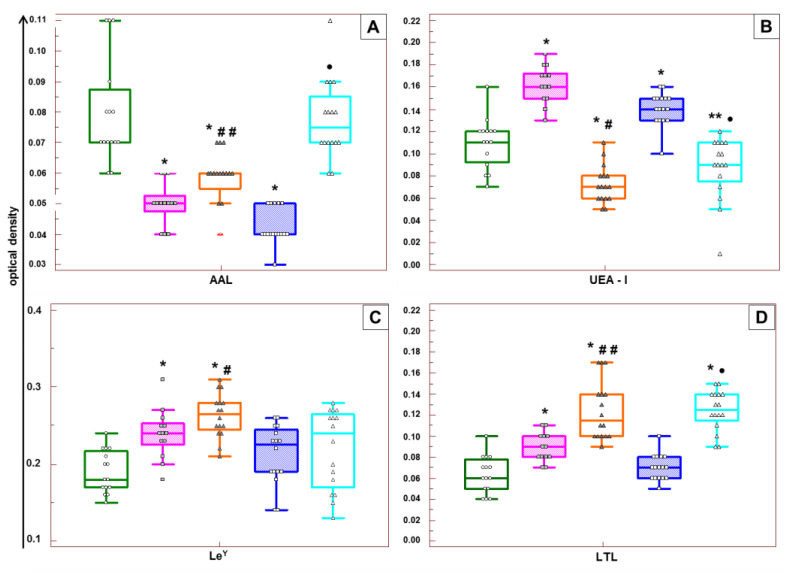
Contents of fucoglycans stained with AAL (**A**), UEA-I (**B**), LTL (**C**), and anti-Le^Y^ antibodies (**D**) in the endothelium of placental terminal villi in patients with normal pregnancy (green boxplots, *n* = 15), in patients with early-onset PE receiving single- (pink boxplots, *n* = 13) or dual-component (orange boxplots, *n* = 16) therapy; and in patients with late-onset PE receiving single- (blue boxplots, *n* = 16) or dual-component (light blue boxplots, *n* = 16) therapy. * Comparison with the level of fucoglycans in the placental samples from patients with normal pregnancy, *p* ≤ 0.0010; ** comparison with the level of fucoglycans in the placental samples from patients with normal pregnancy, *p* = 0.0200; # comparison with the level of fucoglycans in the placental samples from patients with pregnancy complicated by early-onset PE and receiving single-component therapy, *p* < 0.0500; ## comparison with the level of fucoglycans in the placental samples from patients with pregnancy complicated by early-onset PE and receiving single-component therapy, *p* ≤ 0.0010; • comparison with the level of fucoglycans in the placental samples from patients with pregnancy complicated by late-onset PE and receiving single-component therapy, *p* < 0.0001.

In patients with early-onset PE, significant differences in the expression of the endothelial glycocalyx of the capillaries of placental villi of all studied fucosylated glycans were revealed, depending on the applied antihypertensive therapy. A low level of expression of AAL-stained fucoglycans was detected in the endothelium of capillaries of placental villi of the patients with early-onset PE (Figure 2A). However, a comparison of groups 1 and 2 revealed significantly higher levels of fucoglycans in patients with two-component therapy. High expression of fucoglycans stained with UEA-I was found in the capillary endothelium of patients receiving single-component antihypertensive therapy. In contrast, the fucoglycan expression was low in two-component therapy (Figure 2B). Le^Y^ glycan, which is present in the specificity profiles of the UEA-I and LTL lectins, was more expressed in the endothelial glycocalyx of patients with two-component therapy (Figure 2C). Fucoglycans stained by the LTL (Figure 2D) showed the same pattern. Therefore, a significantly higher level of expression of Le^Y^ and LTL- and AAL-stained fucoglycans, but a lower level of fucoglycans stained with UEA-I, were found in the eGC of placental terminal villi of patients with early-onset PE treated with two-component therapy, than in patients taking Dopegyt alone. 

The expression levels of fucoglycans in the eGC of the placental capillary villi at later terms were compared between the groups with normal and complicated pregnancy. The expression of fucoglycans stained with LTL was found to be comparable to a normal level in placenta tissue of patients with single-component therapy (Figure 2D); the findings were similar for fucoglycans stained with anti-Le^Y^ antibodies in the placenta tissue of patients with PE under both regimens of antihypertensive therapy (Figure 2C). Fucoglycans stained with UEA-I showed significantly increased concentrations in the eGC of placental villi in patients with one-component therapy, and decreased levels in patients with two-component therapy (Figure 2B). Relatively high expression of AAL- and LTL-anchored glycans was detected in the endothelium of patients with two-component therapy, with minimal detection of AAL-anchored fucoglycans in single-component therapy (Figure 2A,D). Thus, the expression patterns of fucoglycans in the eGC of placental terminal villi in patients with late-onset PE taking different antihypertensive therapy regimens showed multidirectional changes compared to the normal pattern. In single-component therapy, the pattern was characterized by low expression of AAL-stained fucoglycans and high expression of UEA-I-stained fucoglycans. In two-component therapy, high expression of AAL- and LTL-anchored fucoglycans was combined with low expression of UEA-I-anchored fucoglycans.

**Figure A1:**
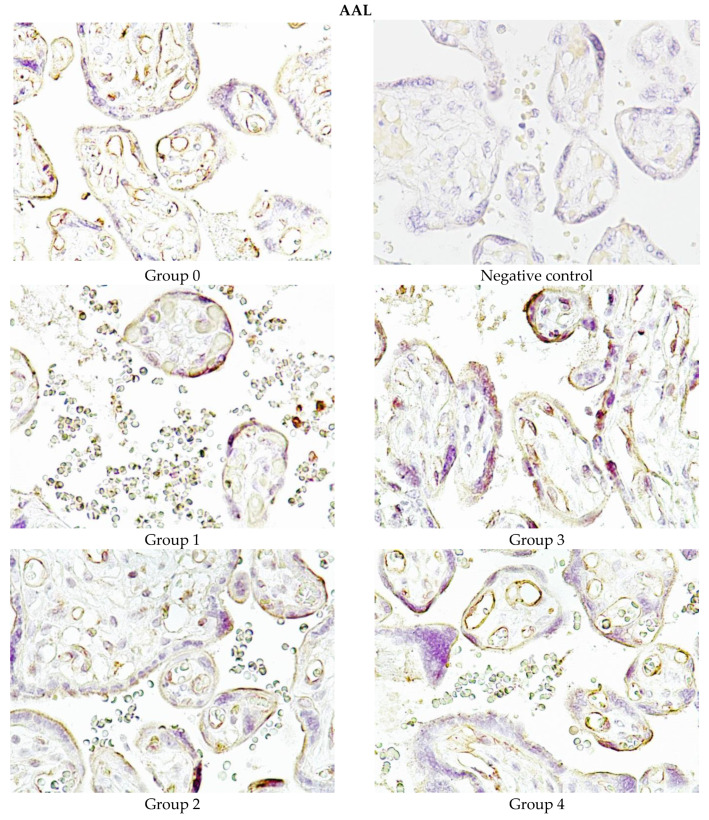
Expression of fucosylated glycans stained with AAL lectin in the endothelial glycocalyx of the capillaries of placental terminal villi. Magnification: ×400.

**Figure A2:**
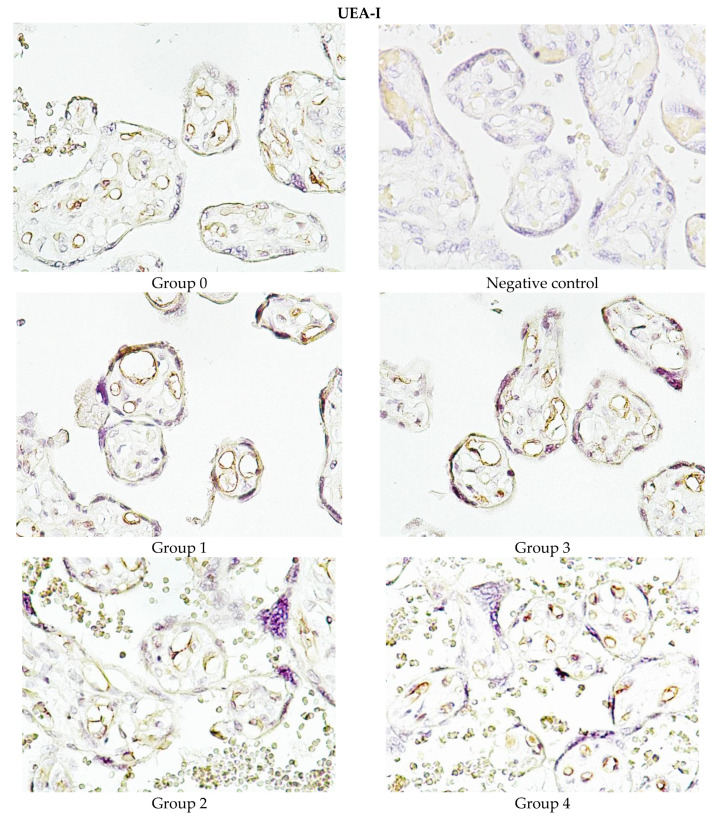
Expression of fucosylated glycans stained with UEA-I lectin in the endothelial glycocalyx of the capillaries of placental terminal villi. Magnification: ×400.

**Figure A3:**
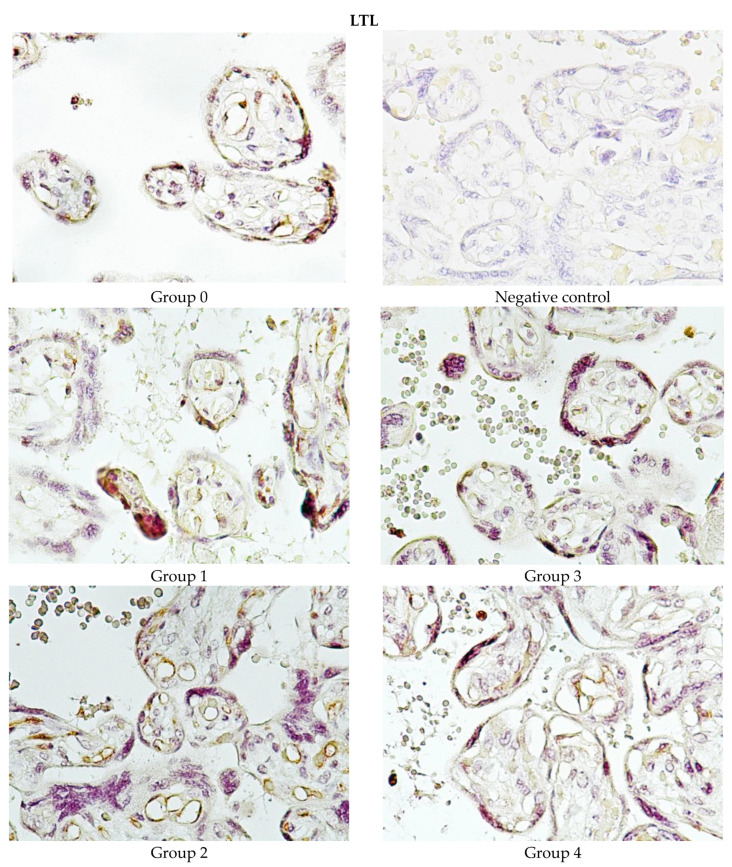
Expression of fucosylated glycans stained with LTL lectin in the endothelial glycocalyx of the capillaries of placental terminal villi. Magnification: ×400.

**Figure A4:**
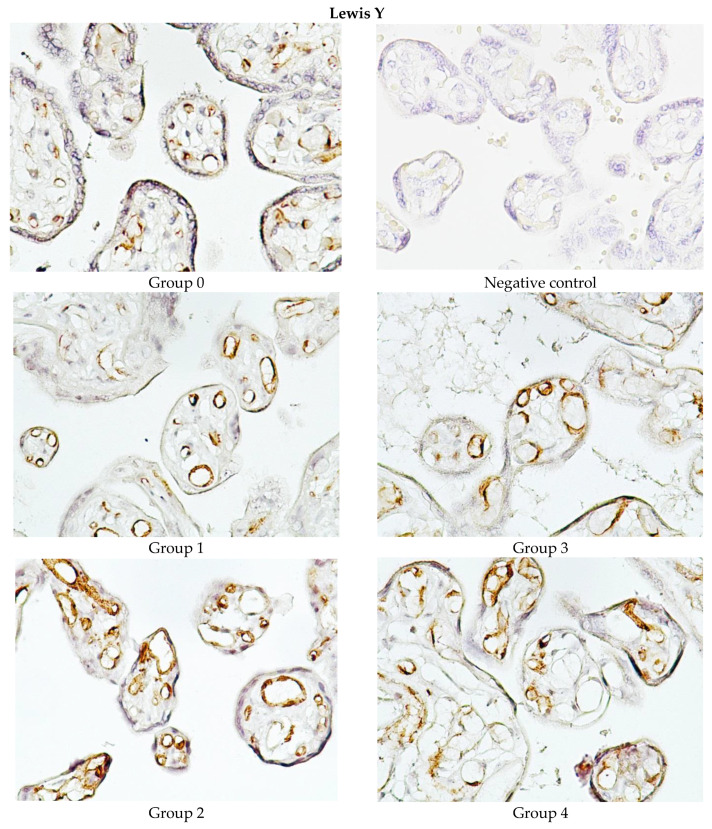
Expression of fucosylated glycans stained with anti-Le^Y^ MAbs in the endothelial glycocalyx of the capillaries of placental terminal villi. Magnification: ×400.

Photomicrographs demonstrating glycoconjugate staining in eGC of fetal capillaries of placental terminal villi are presented in Appendix A (Figure A1, Figure A2, Figure A3 and Figure A4).

### 2.4. Hemodynamic Status of Early- and Late-Onset PE Patients Receiving Single- or Dual-Component Therapy

The analysis of the patients’ hemodynamics showed significant changes in the daily trends of blood pressure, i.e., an increase in the maximum and average values of SAD and DAD in patients with PE with both antihypertensive therapy regimens. The main indicators of arterial stiffness—PWVao and RWTT—as well as the AIx augmentation index, did not show significant changes between the groups (Table 2). At the same time, there was a significant difference in the following indicators between the groups: (dP/dt)_max_, which indirectly reflects myocardial contractility, total stiffness of the main arteries, and dynamic loading; and ED, which reflects the duration of the left ventricular ejection (Table 2). Both indices were elevated compared to Group 0 and also differed in patients at similar gestational term receiving different antihypertensive therapy regimens.

### 2.5. Evaluation of Hemodynamics in the Mother-Placenta-Fetus System

Doppler velocimetry findings (Table 3) showed a significant increase in the mean PI values for uterine arteries, umbilical arteries (but not for fetal cerebral artery), and CPR in all study groups. However, a pairwise comparison revealed that there were no significant differences in CPR and PI of umbilical arteries in patients with either early- or late-onset PE receiving one- and two-component therapy. There were also no differences found in pairwise comparisons for uterine artery PI in women receiving single- or two-component therapy in late-onset PE, or for CPR in early-onset PE (Table 3).

### 2.6. Determination of Cytokines, Soluble Structural Components of Endothelial Glycocalyx, and Associated Proteins in Maternal Blood

Study of the factors reflecting inflammatory reaction in the peripheral blood of the patients (Table 4) demonstrated that the IL-6 level was significantly higher in patients with PE. In addition, the highest cytokine levels were detected in women with PE using two-component therapy. TNFα levels were also elevated in patients with PE, with maximum levels found in patients with late-onset PE.

### 2.7. Correlation Analysis between the Levels of Fucosylated Glycans in the Endothelium of Placental Terminal Villi and Indicators of the Maternal Hemodynamic Profile, Fetoplacental System, and Humoral Factors of Maternal Peripheral Blood

**Figure 3:**
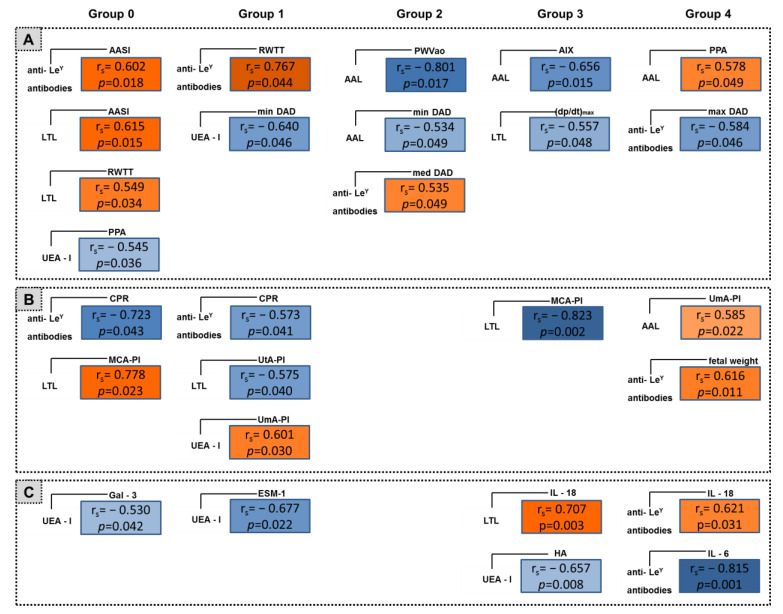
Correlations between the relative levels of fucoglycans stained with lectins and anti-Le^Y^ antibodies in the endothelium of placental terminal villi: and maternal hemodynamic parameters (**A**); fetal Dopplerometry (**B**); and the contents of factors reflecting the inflammatory reaction in peripheral blood (**C**). Group 0—patients with normal pregnancy (*n* = 15); Group 1—patients with early-onset PE receiving single-component therapy (*n* = 13); Group 2—patients with early-onset PE receiving dual-component therapy (*n* = 16); Group 3—patients with late-onset PE receiving single-component therapy (*n* = 16); Group 4—patients with late-onset PE receiving dual-component therapy (*n* = 16).

To identify pathogenetic regularities and trends reflecting the effect of antihypertensive therapy on the expression of fucoglycans in the placenta and the relationship of these expression changes with the disorders of uteroplacental blood flow, maternal hemodynamics, and signs of sterile inflammation in blood, we conducted a study of correlations between the relative levels of glycoconjugates stained with lectins and antibodies, and a complex of indicators reflecting (1) hemodynamic status of the mother (Figure 3A); (2) functioning of the fetoplacental system (Figure 3B); and (3) the signs of sterile inflammation in maternal blood (Figure 3C). The results of correlation analysis were compared between the patients with early-onset PE treated with single-component (Group 1) and two-component (Group 2) antihypertensive regimens; and between patients at later gestational age, with physiological pregnancy (Group 0), with late-onset PE receiving single- (Group 3) and two-component (Group 4) antihypertensive therapy. The results of correlation analysis are presented in Figure 3.

Different patterns of correlations were observed in patients with early-onset PE receiving single-component (Group 1) and two-component (Group 2) therapy (Figure 3). In Group 1, the associations of medium intensity (both direct and reciprocal) between the expression levels of fucoglycans stained with UEA-I, LTL, and anti-Le^Y^ antibodies, and various parameters from the three groups of factors presented in Figure 3, were noted. This suggests related changes in the molecular composition of the eGC of fetal placental vessels and changes in (1) maternal hemodynamics; (2) factors that are the signs of destruction of the eGC, and factors that cause this destruction; and (3) indicators of fetoplacental blood flow (Figure 3A–C). In patients with early-onset PE receiving dual-component therapy, only the associations between the level of glycoconjugates stained with AAL and anti-Le^Y^ antibodies and maternal hemodynamic parameters were found (Figure 3A). Correlation patterns in patients with early-onset PE included strong connection of changes in the expression of fucoglycans, especially fucoglycans with N-acetyllactosamine core (Galβ1-4GlcNAc, type 2 carbohydrate structures), with the changes in blood pressure, arterial stiffness, and destruction of maternal vascular glycocalyx, and with changes in uteroplacental and fetal-placental blood flow in patients with single-component therapy (Figure 3A–C). With two-component therapy, the pattern of correlations includes interrelated changes in the expression of fucoglycans having both core types—isolactosamine Galβ1-3GlcNAc (type 1 structures) and N-acetyllactosamine Galβ1-4GlcNAc (type 2 carbohydrate structures)—with maternal hemodynamic changes, including blood pressure and arterial stiffness parameters (Figure 3A). 

The correlations in later terms of pregnancy (Groups 0, 3, and 4) had different patterns (Figure 3A–C). Patients with one-component therapy predominantly showed reciprocal connections, while patients with two-component therapy, on the contrary, showed direct connections. The patterns of correlations in Group 3 mostly involved the fucoglycans stained with LTL. In particular, reciprocal relationships were found with the pulsation index in the fetal middle cerebral artery and with the hemodynamic index (dP/dt)_max_, which collectively characterizes myocardial function, dynamic load, and arterial stiffness, and is significant for intergroup comparisons. In a normal physiological state, the relationship between the mentioned indicators was also noted, but its character was opposite. Both connections were strong and, apparently, may be pathigenetically significant. Reciprocal relationships of medium strength were found between UEA-I-anchored fucoglycans and hyaluronic acid content in blood, and between AAL-anchored fucoglycans and AIx augmentation index. The only direct relationship in this pattern was found between LTL-anchored glycans and IL-18. 

In contrast, in patients with two-component therapy, the pattern of correlation predominantly showed direct connections. Moreover, fucoglycans stained with anti-Le^Y^ antibodies exhibited both reciprocal connections with the maximum value of DAP, as well as with the IL-6 blood level (strong connection), and direct connections with the IL-18 level and the neonate’s birthweight. AAL-anchored fucoglycans positively correlated with pulse pressure amplification (PPA) and the pulsatile index in the umbilical artery.

## 3. Discussion

Fucosylated glycans are key molecules in intercellular interactions, and are involved in signal transduction in the cell since they are a part of the cell’s receptor apparatus [15,22]. The carbohydrate part of the endothelial cell receptor is a component of the eGC, a structure which determines the main functions of endothelial cells: regulation of vascular tone and vascular permeability, and adhesive interactions with cells and blood proteins. Damage, shedding, and disruption of the eGC structure critically affect the properties of endothelial cells, which change their biochemical response under the shear stress; the outcomes are hemodynamic disorders, capillary leak syndrome, and risk of the formation of blood clots. A number of studies have shown a correlation between eGC destruction and clinical symptoms of PE [23,24,25,26]. In previous studies, we have found association between eGC damage and impaired maternal hemodynamics, and characterized the molecular-functional patterns of vessels in EOPE and LOPE, which confirmed the correlation of circulating components of eGC with indicators of central hemodynamics, arterial stiffness, and myocardial changes [27]. However, eGC has been studied only in part: the majority of eGC glycans are glycosaminoglycans and carbohydrate chains of proteoglycans, but the input of other carbohydrate structures has not been investigated.

Fucosylated glycans, as a functional component of the eGC, have not been studied so far, despite the fact that their expression has been found in endothelial cells, particularly in the endothelium of the fetoplacental system [28,29,30,31,32,33]. Our previous studies suggest that the expression of fucoglycans is different in the endothelium of stem and terminal villi of normal and pathologic placentas [19,34]; this is evidence that their expression is dependent on pathophysiologic factors complicating pregnancy. Hypoxia may be one of these factors, since it (i) results from placental pathology, which is caused by morphologic or occlusive-stenotic malformations of the vessels of fetoplacental complex, and is a characteristic sign of early-onset and late-onset PE; (ii) is both a factor that stimulates the development of sterile systemic inflammation and endothelial dysfunction in response to placental damage [35,36], and a factor destabilizing the eGC [37,38,39], thus leading to hemodynamic disorders; (iii) is one of the key stimulating factors of angiogenesis [40,41], which is impaired in patients with placenta-associated complications of pregnancy, in particular, PE and FGR [42,43]; and (iv) is a regulator of glycan expression in human placental structures [44] (in particular, hypoxia stimulates the expression of fucosylated glycans on the surface of endothelial cells) [45,46]. It should also be noted that, in addition to hypoxia, changes in the expression of glycans, including fucoglycans, activate pro-inflammatory factors [47,48]. Based on the above facts, we suggested that the expression of fucosylated glycans in the eGC of fetal capillaries of the placental terminal villi is connected with maternal and fetoplacental hemodynamic parameters, as well as with the factors reflecting the degree of development of systemic inflammatory response and endothelial dysfunction. Since antihypertensive therapy is a necessary part of the management of patients with PE, we divided the patients according to the applied therapy, which additionally made it possible to assess the differences in the fucoglycan expression under different therapy regimens. 

Histochemical and immunohistochemical studies of the placental tissue revealed similar patterns of changes in the expression of fucosylated glycans in EOPE and LOPE during one-component and two-component antihypertensive therapy; this assumes a similar impact of treatment on the expression of fucoglycans in the eGC of fetal capillaries of placental terminal villi. In particular, at both earlier and later pregnancy terms, the median expression of glycoconjugates stained with AAL, LTL lectins, and anti-Le^Y^ antibodies in patients treated with one-component therapy was lower than that in patients treated with two-component therapy; the opposite effect was observed in UEA-I-stained glycans. In the patients receiving two-component therapy, which was prescribed for more severe and persistent forms of hypertension, the fucoglycan expression in the endothelium of the terminal villi differed slightly only from that in the normal placenta. These results are of significant interest since the evaluation of glycan expression of the fetoplacental system in the eGC during antihypertensive therapy including Dopegyt^®^ and Cordaflex^®^ has not been performed before. 

The specificity of lectins defined with PGA allowed us to determine that the fucoglycans with N-acetyllactosamine core Galβ1-4GlcNAc were predominantly expressed in the glycocalyx of the endothelium of placental terminal villi. The expression (in relative units of optical density) of Le^Y^ stained with anti-Le^Y^ antibodies (specific only to Le^Y^ glycan) was the highest in all groups, while in patients with LOPE it did not differ from normal under both treatment regimens. Despite binding to a similar type of glycan (based on type 2 structures), and the presence of common glycans in the specificity profiles, the binding activity patterns of LTL and UEA-I lectins were different. While UEA-I has a high binding activity, mainly to glycans H type 2, Le^Y^, and a number of sulfated and sialylated N-acetyllactosamine derivatives, LTL has a top specificity to glycans that differ from those detected in the UEA-I profile. Moreover, LTL weakly binds to Le^Y^ and H disaccharide (Fucα1-4GlcNAcβ-). Despite the presence of Le^Y^ in the UEA-I profile, the expression patterns of UEA-I-stained glycoconjugates differ from those detected by staining with anti-Le^Y^ antibodies. Notably, while the expression of UEA-I- stained glycans in the endothelium of fetal capillaries of terminal villi was higher with one-component therapy than with two-component therapy, for the LTL-stained glycans the correlation was opposite. This contrast may be due to the revealed differences in the specificity profiles of the mentioned lectins, and indicates that the variety of glycans with an N-acetyllactosamine-based carbohydrate core, expressed in fetal capillary eGC, is vast and not limited only to H type 2 glycans and the difucosylated oligosaccharide Le^Y^, which expression in PE has been described previously [30,33]. Also of note is a weak staining of placenta tissue with AAL, despite the broad profile of glycans to which it binds on PGA, and the large number of top glycans (i.e., showing high binding activity) in its profile. Moreover, both structures with N-acetyllactosamine core and isolactosamine core (Galβ1-3GlcNAc, type 1 structures) are present in the AAL specificity profile. However, despite the fact that H type 2 and Le^Y^ glycans are present in the AAL binding profile and show high binding activity to this lectin on PGA, the lectin’s target in the tissues of the pathological placenta seems to have a specific composition and structure, which makes the detection of the glycans difficult. Weak binding of AAL may also be due to the lack of expression of fucoglycans with isolactosamine-based core in the eGC of capillaries of placental terminal villi. We found a similar picture in the placental tissues from women whose pregnancies were complicated by FGR: there was no staining of the fetal capillary endothelium with the bacterial lectin BL2LC-Nt, which specifically binds to fucoglycans with type 1 core [19]. 

To interpret the functional significance of the revealed peculiarities of fucoglycan expression, the study of correlations with certain indicators of patient’s hemodynamics, hemodynamics in the fetoplacental system, and the severity of pro-inflammatory response was carried out. Intergroup analysis revealed the signs of activation of systemic inflammatory response (high level of IL-6 and TNFα) in patients with PE treated with antihypertensive drugs. Moreover, in previous studies, we found that pro-inflammatory response was more expressed in patients with LOPE [14]. This is consistent with the studies that suggest a more pronounced activation of the systemic inflammatory response, detected by maternal inflammatory markers, in LOPE than in EOPE [3].

The correlation analysis in each group revealed unique patterns of correlation between the studied factors. In particular, in patients with healthy pregnancy (Group 0), the pattern of correlations suggests that the expression of Le^Y^ and LTL-stained placental fucoglycans regulates maternal and fetoplacental hemodynamics. This regulation may be processed through the maintenance of the optimal expression of fucoglycans, as confirmed by the detected direct correlations with certain maternal hemodynamic parameters, in particular, arterial stiffness (AASI, RWTT) and fetal arterial hemodynamics (MCA-PI). Since an increase in blood flow, assessed by an elevation in MCA-PI, is a compensatory adaptive mechanism in hypoxia [49], and an increase in AASI and RWTT indirectly reflects an augmentation of arterial stiffness, a direct correlation between the fucoglycan expression and these parameters indicates the existence of a mechanism limiting their expression beyond a certain level. This suggestion is indirectly supported by the published data on the increased expression of Le^Y^ (a factor with angiogenic activity in certain pathologic conditions) [50,51], which can be explained as a compensatory effect or a mechanism of placental adaptation to hypoxia [42,52,53].

The study also revealed the reciprocal relationships in Group 0 between UEA-I-stained glycans and (i) the pulse pressure amplification (PPA), which reflects changes in central aortic pressure, and (ii) the blood levels of galectin-3, a carbohydrate-binding protein, which binds to lactosamine and its derivatives (our unpublished data), is associated with glycocalyx [54,55], and is a marker of cardiac remodeling and heart failure [56,57]. These relationships also confirm an important role of maintaining a certain level of fucoglycan expression in the eGC of placental terminal villi.

Correlation analysis for Groups 1 and 2 demonstrated certain differences in the correlation patterns. No correlation patterns were found for healthy pregnancy, but there were many correlations between the fucoglycan expression and maternal hemodynamic parameters, hemodynamics of the fetoplacental system, and the factors associated with the development of inflammatory response in Group 1, while in Group 3 the fucoglycan expression correlated only with maternal hemodynamic parameters. These data, together with a significantly different level of fucoglycan expression in placental tissues of patients in Groups 1 and 2, which was found in intergroup comparisons, indicate various types of regulatory interactions between the studied factors, possibly determined by the type of therapy.

The correlation analysis in Group 3 and Group 4 showed correlation patterns that were different from those in Group 0. Interestingly, in Group 3 a reciprocal strong correlation between the expression level of LTL-stained fucoglycans and MCA-PI was found. Moreover, a similar direct strong correlation was found in Group 0 (discussed above). Taking into account that the expression of LTL-stained fucoglycans in Group 3 was comparable to normal; the reciprocal correlation between the fucoglycan expression and the parameters that depend on arterial stiffness, (dP/dt)_max_; and the strong direct correlation between the fucoglycan expression and the level of IL-18, the pathogenetic significance of these relationships was suggested. It should be noted that in LOPE, correlation patterns in both therapies included markers of inflammation and destabilization of the eGC; these findings confirm a substantial pro-inflammatory response in patients with LOPE, which does not change with antihypertensive therapy.

## 4. Materials and Methods

### 4.1. Selection of Patients for the Study

The interventional longitudinal pilot study included 76 patients. The study was conducted at the National Medical Research Center for Obstetrics, Gynecology, and Perinatology named after academician V.I. Kulakov of the Ministry of Health of the Russian Federation (hereinafter referred to as the Center) in accordance with the principles of the World Medical Association Declaration of Helsinki. The study design was approved by the local ethical committee of the Center (protocol No. 5 of 27 May 2021). All patients signed an informed consent for participation in the study. The main group included 61 patients who were divided by gestational age and type of antihypertensive therapy. A total of 29 patients with early-onset PE (up to 34 weeks) were included: 13 patients receiving one-component treatment with Dopegyt^®^ (Group 1) and 16 patients receiving two-component antihypertensive therapy with Dopegyt^®^ + Cordaflex^®^ (Group 2). A total of 32 patients with late-onset PE (after 34 weeks) were enrolled: 16 patients receiving one-component (Group 3); and 16 patients receiving two-component antihypertensive therapy with Dopegyt^®^ + Cordaflex^®^ (Group 4). The comparison group included 15 pregnant women with physiological pregnancy at 36–39 weeks of gestation (Group 0). 

According to clinical recommendations of the Ministry of Health of the Russian Federation and international protocols, PE is defined as elevation of blood pressure ≥ 140 mmHg systolic and/or 90 mmHg diastolic arterial pressure, emerging at or after 20 weeks of gestation, usually accompanied by proteinuria ≥ 0.3 g/L and/or maternal acute kidney failure, liver dysfunction, neurological signs, hemolysis or thrombocytopenia, and/or fetal growth restriction. Inclusion criterion for the main group was PE, and for the comparison group was healthy pregnancy. Exclusion criteria were pregnancy resulting from ART, severe somatic pathology, history of organ transplantation, immunotherapy during pregnancy. Withdrawal criteria were HELLP syndrome, and acute and chronic infectious and viral diseases during pregnancy. Patients were included in the study using the pair matching method based on age, BMI, and gestational age.

### 4.2. Description of the Antihypertensive Therapy Regimens

Antihypertensive therapy included central-acting medication Dopegyt^®^ (average initial daily dose 750 mg, at later terms 2000 mg). Additional use of prolonged calcium channel blocker medication Cordaflex^®^ (average initial daily dose 40 mg, at later terms up to 120 mg) was prescribed if hypertension persisted despite Dopegyt^®^ at maximal dose intakes. The average duration of therapy in both groups was no less than 16 days. For adjustment of antihypertensive therapy, 24 h ambulatory blood pressure monitoring (ABPM) was performed using a BPLab^®^ device (Petr Telegin LLC, Nizhny Novgorod, Russia), which is recommended for pregnant women [58].

### 4.3. Microarray Chip Analysis

Specificity analysis of Aleuria aurantia lectin (AAL, 2BScientific, cat. no. B-1395-1), Ulex europaeus agglutinin-I (UEA-I, Vector Laboratories, cat. no. B-1065-2), Lotus tetragonolobus lectin (LTL, Vector Laboratories, cat. no. B-1325-2), and primary mouse monoclonal Anti-Blood Group Lewis Y antibody (LWY/1463, cat. no. ab219336, ABCAM) (anti-Le^Y^ MAbs are monoclonal antibodies that recognize difucosylated blood group related antigen Lewis-Y (Le^Y^) [Fucα1-2Galβ1-4(Fucα1-3)GlcNAcβ-R]) was performed on a polymer-coated glass slide with an N-hydroxysuccinimide-derivatized surface, produced by Schott-Nexterion (Jena, Germany), with 651 spacered glycans in 50 μM solutions in 6 replicates, as described in [21]. The main steps of microarray chip analysis included (i) microarray staining with biotinylated lectins; (ii) microarray staining with fluorescent-labeled streptavidin; (iii) obtaining microarray images using a fluorescence reader (intensity of fluorescence reflects interaction between glycan and lectin); and (iv) processing of images using software to obtain quantitative data. Microarray analysis methodology is described in detail by Knirel et al., 2014 [59].

### 4.4. Lectin Histochemistry and Immunohistochemistry of Placental Tissues

Placenta examination was performed on paraffin sections of samples of the paracentral area. Placentas obtained after cesarean section were subjected to macroscopic and microscopic evaluation according to the recommendations of the Amsterdam Consensus [60]. The samples stained with hematoxylin and eosin were microscopically assessed to exclude from the study tissue fragments with hemorrhages, calcificates, and massive fibrinoid deposits.

Lectin histochemistry was performed according to the manufacturer’s protocol “Detection of Glycoproteins Using Lectins in Histochemistry”, Vector Laboratories [61]. Biotinylated lectins were used at concentrations of AAL—5 μg/mL, UEA-I—10 μg/mL, and LTL—20 μg/mL in PBS. The tissues of mature placenta after healthy pregnancy served as positive controls; negative control reactions were performed without adding lectins to the incubation medium.

Immunohistochemical reactions were carried out on paraffin tissue sections using an automated immunohistochemical stainer (Ventana BenchMark XT, Ventana Medical Systems S.A., Kaysersberg, France), according to the manufacturer’s protocol. Detection was performed using the Ventana ultraVIEW DAB Detection kit (Ventana Medical Systems, Inc.). The 2.5 μm tissue sections were deparaffinized using the EZ Prep solution (Ventana Medical Systems, Inc.; cat. no. 05279771001). Heat-induced antigen retrieval was performed using the Cell Conditioning 1 solution (Ventana Medical Systems, Inc.; cat. no. 05424569001) at 95 °C for 30 min. Endogenous peroxidase activity was blocked by treatment with the ultraVIEW inhibitor (Ventana Medical Systems, Inc.; cat no. 05269806001) in 3% H_2_O_2_ for 4 min.

The slides were incubated with the anti-Le^Y^ MAbs (2.5 µg/mL) for 64 min. The ultraVIEW Universal DAB Detection kit incorporates multimer technology, whereby the ultraVIEW streptavidin horseradish peroxidase (HRP) enzyme is directly conjugated to the secondary antibody. 

Slides were incubated with a secondary antibody of ultraVIEW HRP Multimer (Ventana Medical Systems, Inc.; cat. no. 05269806001) at 37 °C for 8 min and a diaminobenzidine + H_2_O_2_ substrate for 8 min, which was followed by counterstaining with hematoxylin II (Ventana Medical Systems, Tucson, AZ, USA, cat. no. 05277965001) and bluing reagent (Ventana Medical Systems, Tucson, AZ, USA, cat. no. 05266769001) for 2 and 3 min, respectively. Slides were washed with Tris buffer (pH 7.6) and mounted using a xylene-based mounting media.

Stained lung carcinoma slices were considered as a positive control, while reactions without anti-Le^Y^ MAb antibody were considered as a negative control.

Quantitative evaluation of the results of lectin histochemistry and immunohistochemistry was performed by measuring the optical density of the stained products in the endothelial membrane of the capillaries of the placental terminal villi. To obtain representative data, measurements were performed in 10 randomly selected equally distant fields of view. A Nikon eclipse 80i light microscope, Nikon DS-Fi1 digital camera (Nicon Corporation, Tokyo, Japan) and NIS Elements Advanced Research 4.1 image analysis software were used for image analysis.

### 4.5. Evaluation of Hemodynamics

The oscillometric method of BP measurement was applied. Twenty-four-hour BP monitoring was performed using the BPLab^®^ monitor (Petr Telegin LLC, Nizhny Novgorod, Russia), which meets international standards of accuracy for oscillometric BP recorders and is recommended for pregnant women [58]. Oscillograms were analyzed using Vasotens software, BPLab^®^ V.06.02.00 (Nizhny Novgorod, Russia). Mean central systolic and diastolic BP were measured during the day and night time, within 24 h. Regular intervals between measurements were 30 min at daytime and 60 min at night. The parameters characterizing changes in blood pressure (BP) were determined: max DAD; max SAD; min DAD; max SAD; med DAD; med SAD. Parameters determined by Vasotens software, BPLab^®^ V.06.02.00 were similar to those used in the study by [27] and included the following:

1. Reflected wave transit time (RWTT): the return time of the wave reflected from aorta;
2. Aortic pulse wave velocity (PWVao): the velocity at which blood pressure pulse propagates into the aorta;
3. Augmentation index (AIx): a noninvasive measure of pulse wave reflection;
4. Arterial stiffness index (AASI);
5. Ambulatory rigidity index (AASI = 1 − (inclination BPdiastolic − BPsystolic));
6. Maximal BP increase velocity ((dP/dt)_max_): indirectly represents myocardial contractility, total vascular resistance, and dynamic load of pulse wave on vascular walls;
7. Pulse pressure amplification (PPA): the increase in pulse pressure (PP) amplitude when pressure waves propagate distally in the systemic network, accompanied by morphological alterations of pressure waveforms;
8. Ejection duration (ED): an interval of blood flow from the start of pulsation till the closure of the aortic valve;
9. Subendocardial viability ratio (SEVR), defined as diastolic to systolic pressure–time integral ratio.

### 4.6. Arterial Doppler

Dopplerometry of maternal–placental and fetoplacental blood flows was performed using transabdominal transducer of the expert ultrasound machines (The Voluson E8 ultrasound system, GE Healthcare Austria GmbH&Co OG, (Tiefenbac, Austria)). Uterine arteries (UtA), umbilical artery (UA), middle cerebral artery (MCA), and ductus venosus pulsatility index (PI), as well as cerebroplacental ratio, were measured. When evaluating umbilical artery PI, positive or absent diastolic flow was assessed. Ductus venosus was analyzed through the cross-section and sagittal plane of the fetal abdomen using Doppler color flow mapping. The curve was defined as normal if the A-wave was positive, or abnormal in case of absent or negative A-wave. 

### 4.7. Determination of Soluble Factors in Maternal Blood

Blood sampling was performed after fasting. Serum samples were collected into vacuum blood-collecting tubes, S-Monovette^®^ Serum, 4.9 mL, cap white, (L×Ø): 90 × 13 mm. Serum preparation for the study was performed according to the standard operating procedure of the Center’s Biobank, where the samples were stored at −80 °C. The study of humoral factors was carried out via the ELISA method using commercial test systems for the determination of cytokines: IL-18 (BMS267-2, Bender MedSystem GmbH, Wien, Austria), IL-6 (A-8768, Vector Best, Novosibirsk, Russia), TNFα (BMS223-4, Bender MedSystem GmbH, Wien, Austria), and galectin-3 (BMS279-4, Bender MedSystem GmbH, Wien, Austria). Determination of desquamated structural components of eGC was performed on test systems manufactured by Cloud-Clone Corp., Katy, TX, USA: SEC463Hu (determination of endocan-1); SEB966Hu (determination of syndecan-1); CEA182Ge (determination of hyaluronic acid).

### 4.8. Statistical Analysis

The software used for statistical analysis was “Statistica 10.0” (StatSoft, Inc., Tulsa, OH, USA) and “MedCalc version 16.4” (MedCalc Software Ltd, Ostend, Belgium). Microarray data are presented using descriptive statistics. The data were processed using ScanArray Express 4.0 (PerkinElmer Life & Analytical Sciences, Shelton, CT, USA) with measurement of median of relative fluorescent units (RFU) of 6 replicates of each glycan on the microarray. The median deviation was measured as an interquartile range. A signal with fluorescence intensity 5 times exceeding the background value was considered significant as described in [62].

The evaluation of clinical, (immuno)histochemical, and biochemical data was conducted using non-parametric methods, since the assessments of skewness and kurtosis in the analyzed distributions significantly deviated from zero. The Kolmogorov–Smirnov test denied the normality of the majority of the distributions. The data are presented as median (minimum, maximum value); the differences in comparisons of three or more groups by a quantitative indicator, whose distribution differed from normal, were calculated using the Kruskal–Wallis analysis of variance with Bonferroni correction (*p* < 0.025). This method was used to compare three groups in later terms of pregnancy. A posteriori comparisons were performed using the Mann–Whitney U test. For pairwise comparisons of data in earlier terms of pregnancy, only the Mann–Whitney U test was used. Differences between parameters were considered significant at *p* < 0.05.

## 5. Conclusions

The patterns of expression of fucosylated glycans in the eGC of the capillaries of placental terminal villi of patients with EOPE and LOPE have a specific fucoglycan composition, with the predominant expression of fucoglycans with type 2 core, which are detected by lectins with different carbohydrate specificity profiles. Regardless of the fucoglycan composition, their expression patterns in the eGC show similar quantitative changes at earlier and later terms of pregnancy with PE, which, apparently, is due to the similar effects of one-component and two-component antihypertensive therapy. The correlation patterns indicate interrelated changes in the molecular composition of eGC fucoglycans in fetal capillaries of the terminal villi and indicators of the maternal hemodynamics, fetoplacental hemodynamics, and humoral factors associated with eGC damage. The present study is the first to demonstrate the features of placental eGC in women with PE treated with antihypertensive therapy; the study also considers placental fucoglycans as a functional part of the eGC, which has an influence on the hemodynamics in the mother–placenta–fetus system.

## Figures and Tables

**Table 1:** Clinical characteristics of the patients included in the study.

Parameter	Group 0 (NP) (*n* = 15)	Group 1 (*n* = 13)	Group 2 (*n* = 16)	Group 3 (*n* = 16)	Group 4 (*n* = 16)	*p*-Level
Age, years	34.0 (28–43)	32.0 (23–44)	34.0 (23–41)	31.5 (26–42)	30.5 (23–42)	*p* ^1^ = 0.6330
SAD med, mm Hg	119 (108–126)	145 (131–154)	143 (136–157)	141 (131–154)	137 (132–148)	***p* ^1^ < 0.0001**
DAD med, mm Hg	75 (70–82)	94 (84–105)	100 (92–109)	101 (91–105)	91 (91–102)	***p* ^1^ < 0.0001**
BMI, kg/m^2^	27.0 (23.0–31.0)	24.0 (19.0–34.3)	25.0 (20.0–51.0)	27.0 (20.0–42.0)	27.0 (17.0–51.0)	*p* ^1^ = 0.3184
Gestational age, weeks	37.0 (34.0–39.0)	30.1 (26.1–33.3)	30.35 (25.3–33.4)	37.55 (36.0–39.3)	37.0 (34.0–40.3)	***p* ^1^ < 0.0001** *p* ^2^ = 0.5982 *p* ^3^ = 0.4644
Mean dose of medication used, mg	-	1500	2000 + 120	1000	2000 + 80	-
Newborn weight, gramms	3310 (2485–3948)	1220 (670–1840)	1335 (440–2300)	2981 (2130–3777)	2726 (1770–3920)	***p* ^1^ < 0.0001**
Apgar 1, score	8 (7–8)	7 (4–7)	7 (2–8)	8 (8–8)	8 (6–8)	***p* ^1^ < 0.0001**
Apgar 5, score	9 (8–9)	8 (6–8)	8 (5–9)	9 (8–9)	8 (7–9)	***p* ^1^ = 0.0001**

*p* ^1^—significance level calculated by the Kruskal–Wallis H test. *p* ^2^—significance level calculated by the Mann–Whitney U test when comparing patients receiving antihypertensive therapy for EOPE. *p* ^3^—significance level calculated by the Mann–Whitney U test when comparing patients receiving antihypertensive therapy for LOPE. Data are presented as median (minimum, maximum value).

**Table 2:** Hemodynamic parameters of the study patients.

Parameter	Group 0 (NP) (*n* = 15)	Group 1 (*n* = 13)	Group 2 (*n* = 16)	Group 3 (*n* = 16)	Group 4 (*n* = 16)	*p*-Level
AASI	0.516 (0.213–0.810)	0.415 (0.313–0.651)	0.436 (0.179–0.729)	0.585 (0.162–0.712)	0.526 (0.170–0.836)	0.7172
AIx, %	−56 (−72–(−9))	−52 (−71–(−19))	−43 (−71–(−13))	−46 (−75–(−12))	−40.5 (−71–(−3))	0.3593
(dP/dt)_max_, mm Hg/s	352 (173–598)	486 (352–681)	561 (173–861)	590 (352–973)	469 (246–811)	**0.0017**
ED, ms	271 (235–304)	343 (292–409)	304 (136–381)	336 (273–398)	322.5 (125–382)	**0.0008**
PPA, %	117 (100–189)	137 (113–189)	129 (107–151)	133 (114–146)	132 (30–144)	0.5558
PWVao, m/s	7.1 (5.8–9.8)	6.8 (5.6–8.1)	7.0 (5.8–7.8)	7.2 (5.0–9.4)	7.3 (5.8–8.2)	0.7586
RWTT, m/s	127 (105–165)	134 (105–136)	130 (105–155)	128 (103–189)	134 (99–180)	0.9402
SERV, %	101 (31–294)	102.5 (31–196)	129 (51–310)	119 (101–152)	116 (98–144)	0.1516
DAD max, mm Hg	81 (74–89)	108 (86–120)	108.5 (102–130)	105 (87–141)	100.5 (92–112)	**<0.0001**
SAD max, mm Hg	129 (124–139)	159 (134–179)	169 (142–194)	162 (146–179)	149.5 (137–188)	**<0.0001**
DAD min, mm Hg	69 (59–73)	74 (57–80)	76 (49–89)	76 (46–83)	63.5 (48–85)	0.1091
SAD min, mm Hg	105 (97–110)	112 (87–124)	113.5 (89–129)	110 (90–127)	105.5 (88–129)	0.0939

*p*—significance level calculated by the Kruskal–Wallis H test. AASI, arterial stiffness index; AIx, augmentation index; (dP/dt)_max_, maximal blood pressure increase velocity; ED, ejection duration; max DAD, maximal aortal diastolic blood pressure; min DAD, minimal aortal diastolic blood pressure; min SAD, minimal aortal systolic blood pressure; med SAD, mean aortal systolic blood pressure; med DAD, mean aortal diastolic blood pressure; max SAD, maximal aortal systolic blood pressure; PPA, pulse pressure amplification; RWTT, reflected wave transit time; PWVao, aortic pulse wave velocity; SEVR, subendocardial viability ratio.

**Table 3:** Mean velocity indices of blood flow in uterine, umbilical cord, and fetal cerebral arteries.

Parameter	Group 0 (NP) (*n* = 15)	Group 1 (*n* = 13)	Group 2 (*n* = 16)	Group 3 (*n* = 16)	Group 4 (*n* = 16)	*p*-Level
UtA-PI	0.76 (0.55–0.91)	1.40 (1.01–1.9)	1.18 (0.48–1.98)	1.00 (0.50–2.55)	1.12 (0.04–2.13)	***p* ^1^ = 0.0010** ***p* ^2^ = 0.0369** *p* ^3^ = 0.6945
UmA-PI	0.86 (0.63–1.19)	1.31 (0.81–2.00)	1.15 (0.91–3.10)	0.98 (0.71–1.09)	0.86 (0.60–1.36)	***p* ^1^ < 0.0001** *p* ^2^ = 0.2442 *p* ^3^ = 0.2216
MCA-PI	1.51 (1.20–1.96)	1.58 (0.93–2.30)	1.67 (1.13–2.63)	1.67 (1.31–2.18)	1.40 (1.25–1.55)	*p* ^1^ = 0.0990 *p* ^2^ = 0.2345 ***p* ^3^ = 0.0064**
CPR	2.06 (1.71–2.55)	0.90 (0.59–1.95)	1.63 (0.39–2.74)	1.67 (1.10–2.41)	1.58 (0.96–2.38)	***p* ^1^ = 0.0005** *p* ^2^ = 0.1077 *p* ^3^ = 0.4491

UtA-PI—uterine artery mean pulsatility index; UmA-PI—umbilical artery pulsatility index; MCA-PI—fetal middle cerebral artery pulsatility index; CPR—cerebro–placental ratio. *p* ^1^—significance level calculated by the Kruskal–Wallis H test; *p* ^2^—significance level calculated by the Mann–Whitney U test when comparing patients receiving antihypertensive therapy for EOPE; *p* ^3^—significance level calculated by the Mann–Whitney U test when comparing patients receiving antihypertensive therapy for LOPE. Data are presented as median (minimum, maximum value).

**Table 4:** Concentration of cytokines, glycans, and associated proteins in maternal peripheral blood.

Parameter	Group 0 (NP) (*n* = 15)	Group 1 (*n* = 13)	Group 2 (*n* = 16)	Group 3 (*n* = 16)	Group 4 (*n* = 16)	*p*-Level
Galectin-3, ng/mL	15.17 (7.83–26.77)	13.76 (6.51–40.73)	12.97 (8.37–26.03)	13.09 (9.27–24.43)	14.82 (9.39–83.24)	*p* = 0.8444
IL-18, pg/mL	46.26 (14.52–242.10)	80.82 (22.41–161.60)	77.64 (12.50–195.05)	75.49 (15.40–176.12)	81.86 (24.39–207.05)	*p* = 0.1930
IL-6, pg/mL	0.09 (0.05–3.20)	2.58 (0.09–58.55)	3.17 (0.09–207.30)	2.81 (0.09–45.74)	3.95 (0.10–569.37)	***p* = 0.0021**
TNFα, pg/mL	0.33 (0.27–0.37)	0.34 (0.33–0.41)	0.34 (0.31–0.39)	0.36 (0.29–2.80)	0.37 (0.33–2.40)	***p* = 0.0111**
ESM-1, ng/mL	0.05 (0.04–0.15)	0.06 (0.04–1.01)	0.07 (90.04–0.13)	0.05 (0.04–0.14)	0.07 (0.04–0.13)	*p* = 0.0662
HA, ng/mL	175.89 (57.62–381.60)	124.69 (51.67–389.97)	99.10 (6.74–376.68)	185.23 (41.03–513.07)	115.23 (4.03–343.38)	*p* = 0.6241
SDC-1, pg/mL	1.29 (0.70–20.16)	3.29 (1.03–26.68)	1.72 (0.79–11.80)	5.72 (1.25–15.22)	2.57 (0.96–8.36)	*p* = 0.2555

*p*—significance level calculated by the Kruskal–Wallis H test. Data are presented as median (minimum, maximum value).

## Data Availability

The datasets generated during and/or analyzed during the current study are available from the corresponding author on reasonable request.
